# Effects of Land Use/Cover Changes and Urban Forest Configuration on Urban Heat Islands in a Loess Hilly Region: Case Study Based on Yan’an City, China

**DOI:** 10.3390/ijerph14080840

**Published:** 2017-07-26

**Authors:** Xinping Zhang, Dexiang Wang, Hongke Hao, Fangfang Zhang, Youning Hu

**Affiliations:** 1 College of Forestry, Northwest A&F University, Yangling 712100, China; jhonxinping81@nwsuaf.edu.cn (X.Z.); hhk_2008@126.com (H.H.); huyouning@163.com (Y.H.); 2 Tourism Department, Shaanxi Vocational & Technical College, Xi’an 710100, China; 3 Gaoling Branch School, Shaanxi Agricultural Broadcasting and Television School, Xi’an 710200, China; yuanyi0918@163.com; 4 School of Biological and Environmental Engineering, Xi’an University, Xi’an 710065, China

**Keywords:** land surface temperature, landscape pattern analysis, spatial random point analysis, single-channel algorithm, urban green space cooling island

## Abstract

In this study Yan’an City, a typical hilly valley city, was considered as the study area in order to explain the relationships between the surface urban heat island (SUHI) and land use/land cover (LULC) types, the landscape pattern metrics of LULC types and land surface temperature (LST) and remote sensing indexes were retrieved from Landsat data during 1990–2015, and to find factors contributed to the green space cool island intensity (GSCI) through field measurements of 34 green spaces. The results showed that during 1990–2015, because of local anthropogenic activities, SUHI was mainly located in lower vegetation cover areas. There was a significant suburban-urban gradient in the average LST, as well as its heterogeneity and fluctuations. Six landscape metrics comprising the fractal dimension index, percentage of landscape, aggregation index, division index, Shannon’s diversity index, and expansion intensity of the classified LST spatiotemporal changes were paralleled to LULC changes, especially for construction land, during the past 25 years. In the urban area, an index-based built-up index was the key positive factor for explaining LST increases, whereas the normalized difference vegetation index and modified normalized difference water index were crucial factors for explaining LST decreases during the study periods. In terms of the heat mitigation performance of green spaces, mixed forest was better than pure forest, and the urban forest configuration had positive effects on GSCI. The results of this study provide insights into the importance of species choice and the spatial design of green spaces for cooling the environment.

## 1. Introduction

It is well known that urbanization is one of the most powerful and visible anthropogenic forces on Earth [1,2,3]. The most obvious aspect of urbanization is that the natural landscape is increasingly being replaced by anthropogenic land use/land cover (LULC) types, which can lead to many ecological and environmental problems, such as urban heat islands [4,5]. In China, cities are expanding rapidly as the economy grows, but land suitable for development is in short supply, particularly in mountainous areas, where about one-fifth of the population lives. Thus, in the last decade, local governments have begun removing the tops of mountains to fill valleys and create land suitable for building. In cities such as Chongqing, Shiyan, Yichang, Lanzhou and Yan’an, tens of square kilometers of land have been created. However, in the Yan’an city area, the relatively warmer urban land surface/atmosphere compared with the rural surroundings is very obvious in the summer, which is usually referred to as an urban heat island (UHI).

### 1.1. Related Research

UHIs were identified for the first time in London during 1833 [6]. An UHI is more obvious under calm, cloudless conditions, where it depends greatly on the urban morphology and land use type [7,8,9]. UHIs are now considered major environmental problems in the 21st century [7,10,11], because the higher temperatures in UHIs can lead to increases in urban energy consumption [12,13,14,15], raised pollution levels [16,17,18], and they may even affect the habitability of cities and increase mortality [12,15,19]. Therefore, methods for mitigating UHIs are now a major research focus. UHIs exhibit different forms associated with at least four spatial regions: boundary-layer UHI (BLUHI), canopy layer UHI (CLUHI), surface UHI (SUHI), and subsurface UHI (SubUHI). The urban canopy layer extends upward from the surface to approximately the mean building height, whereas the urban boundary layer is located above the canopy layer [20]. CLUHI and BLUHI are atmospheric heat islands caused by warming of the urban atmosphere, whereas SUHI refers to the relatively higher warmth of urban surfaces compared with surrounding rural areas. SubUHI is part of the overall UHI, which denotes the relative warmth of urban ground temperatures compared with the rural background [21]. It is known that atmospheric UHIs are larger at night whereas surface UHIs are larger during the day [22]. SUHIs have stronger effects on the daily outdoor activities of people. Moreover, SUHIs can be measured conveniently at different spatial-temporal scales and simulated based on remote sensing data. The effects of SUHIs can be evaluated using air temperature measurements and satellite land surface temperature (LST) measurements. The air temperature measurements are obtained either by traversing a city or by comparing point temperature measurements [23,24]. In general, SUHI measurements based on air temperature have high temporal resolution with extensive time coverage, and they can effectively describe the temporal variation in the effects of UHIs [25]. However, spatially continuous analysis is often difficult because of the sparse distribution of observation stations. Fortunately, the LST is a universal and important parameter for analyzing SUHIs. To address these problems, many studies of the effects of SUHIs have considered LST data, which were mainly measured using two approaches. Traditionally, LST data are measured via ground-based observations obtained from automobile transects and weather station networks [20]. However, at present, due to the development of thermal remote sensors, satellite-based imaging technology is now employed widely to detect UHIs remotely and regionally because it facilitates the straightforward and consistent determination of the spatial-temporal LST distribution [26]. Thus, AVHRR and ATSR [27,28], Moderate Resolution Imaging Spectoradiometer (MODIS) [29], Landsat Thematic Mapper (TM) [30,31], Landsat Enhanced Thematic Mapper Plus (ETM+) [32], Landsat Operational Land Imager and Thermal Infrared Sensor (OLI & TIRS) [33,34], Chinese HJ-1B Infrared Multispectral Scanner (IRMSS) [35,36,37] alone or combined with high-resolution satellite images, such as SPOT [38], Gaofen-1 [29], or Quickbird [25,39,40,41], can be used for the rapid retrieval of LST data. In addition, hyperspectral thermal infrared data, such as those acquired by the Infrared Atmospheric Sounding Interferometer (IASI) and Cross-track Infrared Sounder (CrIS), have been used for retrieving LST data [42]. Therefore, LST data derived from thermal infrared remote sensors are among the most commonly used indicators for heat island analysis. In the last two decades, many studies have focused on identifying the factors that contribute to SUHIs [7,10,24,43,44,45,46,47], which have shown that urban built-up land and the impervious surface area have positive effects on SUHIs [48], whereas water bodies and green landscape have negative effects on SUHIs [24,43,49,50]. In addition, previous studies have demonstrated that there are complex relationship between the landscape composition [25,39,44], land use and cover changes (LUCC) [3,36,51,52], and SUHIs.

Urban green spaces (UGS), including urban parks, green belts, attached green space, and productive plantation areas, are considered to be important components of urban vegetation, where they are cooler than their surrounding built-up areas and they can form a “green space cool island” (GSCI).

A previous study suggested that the GSCI intensity is affected by the size, shape, seasonal changes (summer/autumn), and the forest structure (stem density, diameter, tree height, basal area, leaf area index (LAI), and canopy density) for urban parks in Shenyang, China [24]. The urban landscape configuration (LULC types and landscape pattern metrics) also influences SUHIs [43,53]. However, the relationships between the GSCI intensity and other aspects of urban forest structures (e.g., species composition, three-dimensional green biomass (TGB), and health) in UGSs have rarely been studied and they are not yet fully understood. Furthermore, previous studies of the spatiotemporal distribution and factors that affect SUHIs have mainly considered municipalities, provincial capitals, or coastal cities, whereas few studies have investigated cities at the prefecture level in the loess hilly region of China.

### 1.2. Study Objectives

In this study, based on field surveys and Landsat series (5/7/8) satellite remote sensing data (1990–2015, 5-year intervals) acquired for Yan’an City, China, we investigated the effects of the LUCC and UGS tree-layer structure on the SUHI intensity. The aims of this study were: (1) to explore the quantitative relationships between the GSCI intensity and UGS structure, and to determine whether the UGS structure significantly affects the GSCI intensity; (2) to examine the spatiotemporal trends in the LST and LUCC during the last 25 years (1990–2015); and (3) to identify the main LULC types that significantly affected the LST during six research periods. The results of this study should be useful for urban planners and designers by facilitating the design of UGSs that maximize the GSCI intensity and mitigate UHIs.

## 2. Materials and Methods

### 2.1. Study Area

**Figure 1:**
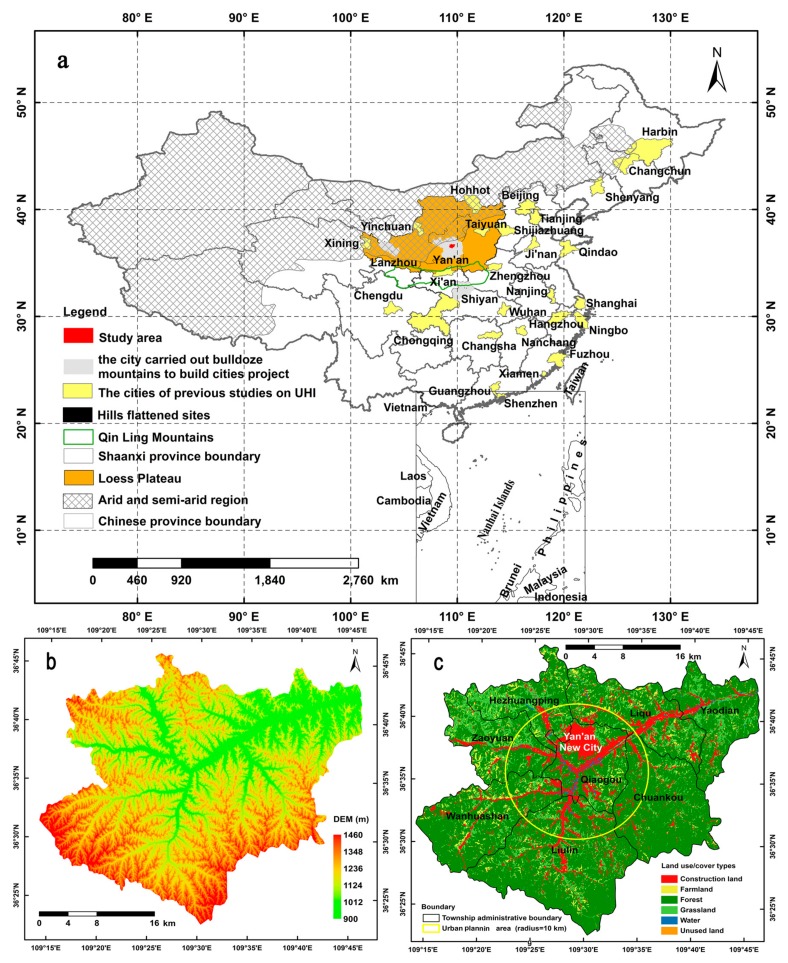
Map of the study area showing: (**a**) its location in China; (**b**) the topographical status; and (**c**) the township administrative boundary (black polyline) and urban planning area (yellow circle) in 2015, where the circle at the center marks the intersection of three mountains (Baota mountain, Qingliang mountain, and Fenghuang mountain), as well as two rivers (Yanhe river and Duchuanhe river). Urban area (UA) is a kind of the construction lands within the urban planning area. The suburban area (SUA) is the area between the exterior of the urban area and the external outline of the study area, in the corresponding period, based on master planning and land use planning of Yan’an city from Baota District Branch of Yan’an Municipal Bureau of Land and Resources.

The case study area, in the Baota District of Yan’an City (36°22′44″–36°45′53″ N, 109°14′7″–109°46′9″ E) in the middle reaches of the Yellow River, where it is located in an arid and semiarid region of the Loess Plateau in Northwestern China, has been affected by severe soil erosion which makes this district a key region for ecological restoration. The case study area covers 1174.54 km^2^ (comprising 1600 × 1432 grid cells at 30-m resolution) and the elevation ranges from circa 900 m to circa 1466 m above sea level (Figure 1). In 2013, this district had a population of 478,500 (Shaanxi Statistical Yearbook in 2014), 63% of whom were rural. This region has a typical semiarid continental climate, with average annual rainfall of approximately 470 mm (with high variability in recent years), where over 65% occurs between June and September, mainly in the form of heavy rain (Baota Meteorological Observatory). The study area contains 260 villages according to the Second National Land Survey of China. This area has undergone long-term soil and water conservation, and it was selected as one of the pioneer demonstration areas for the large-scale ecological restoration project known in China as “Grain to Green” [4,54,55].

Petroleum exploitation has been conducted throughout the study area by the Shaanxi Yanchang Petroleum (Group) Co. Ltd. (Yan’an, China). During the past three decades, and road construction on forestland or farmland in order to transport the crude oil from exploitation sites to oil refineries has destroyed large amounts of vegetation.

One of the largest construction projects in the study area, which started in April 2012 (Figure 1), doubled the city’s current area by creating 78.5 km^2^ of flat ground [56], on which the new Yan’an City was built. Most of the vegetation was removed from more than 30 hills.

**Figure 2:**
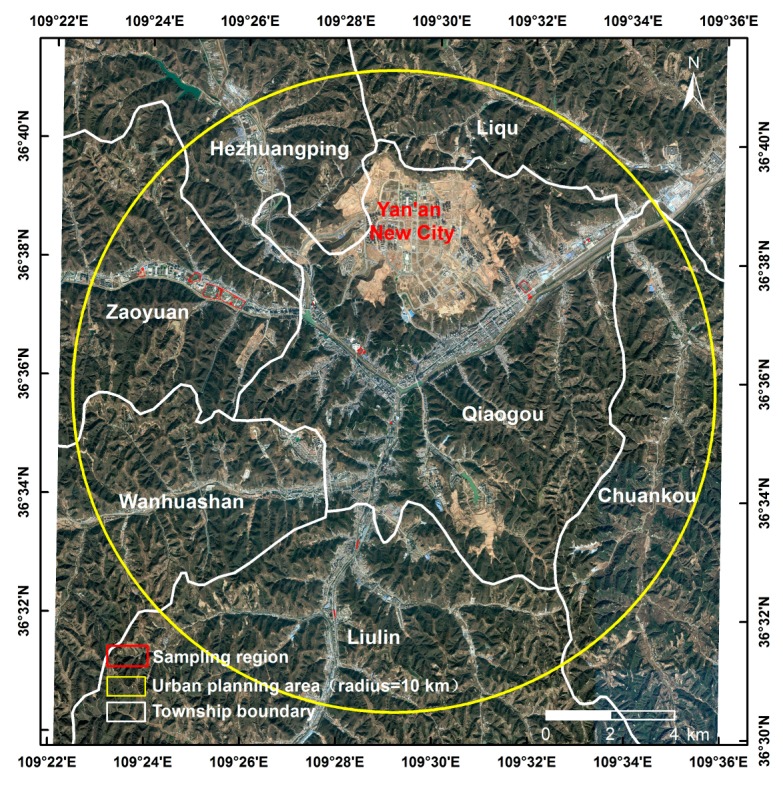
Map showing the distribution of sampling points in the region (plots).

According to forest resource inventory data (2006) and field surveys (July to August, 2012–2015), the dominant tree species in the study area are: *Robinia pseudoacacia* Linn., *Platycladus orientalis* (L.) Franco, *Quercus wutaishansea* Mary, *Pinus tabuliformis* Carr., *Betula platyphylla* Suk., *Populus davidiana* Dode., *Pyrus betulifolia* Bunge, *Ulmus pumila* L., *Malus pumila* Mill., *Juglans regia* L., and *Ostryopsis davidiana* Decne (Figure 2). The soil in the study area is mainly loessial soil according to Loess Plateau soil data [57].

### 2.2. Data Sources

In this study, three types of data were utilized: (1) Baota district meteorological data (Baota district bureau of meteorology), (2) Baota district land use data (provided by Baota District Branch of Yan’an Municipal Bureau of Land and Resources), and (3) Landsat satellite images (path/row: 127/35), downloaded from the United States Geological Survey [58]. Further details of these data are given in Table 1.

### 2.3. Methods

#### 2.3.1. Technical Details

**Figure 3:**
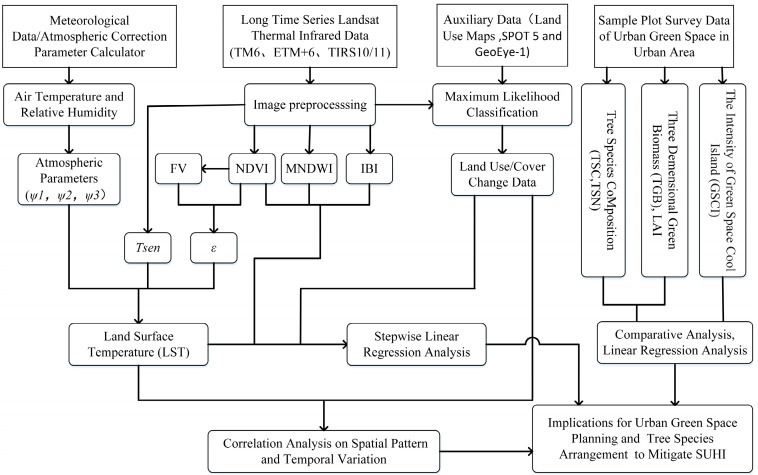
Methods employed in this study, where the definitions of variables are given in Equations (1)–(19). *ψ*: atmospheric parameters; FV: vegetation fraction; NDVI: normalized difference vegetation index; MNDWI: modified normalized difference water index; IBI: an index-based built-up index; Tsen: at-sensor temperature; ε: land surface emissivity; SUHI: surface urban heat island.

The present study of the changes in the fraction of suburban vegetation and urban green spaces (UGSs), and their effects on the thermal environment considered four main issues (Figure 3): (1) the fraction of suburban vegetation and the characteristic changes in UGSs were studied using spatial analysis and statistical analysis based on land use and cover change (LUCC) and associated remote sensing indicators; (2) the characteristic changes in the thermal environmental were examined using the land surface temperature (LST) single-channel algorithm and the landscape assessment method; (3) the effects of LUCC on the thermal environment were analyzed using a spatial linear regression method for the LST data versus land surface remote sensing indicators; and (4) the mitigating effects of UGSs on surface urban heat islands (SUHIs) were assessed based on urban forest configuration-related indicators.

#### 2.3.2. Derivation of the Normalized Difference Vegetation Index (NDVI), Index-Based Built-Up Index (IBI), and Modified Normalized Difference Water Index (MNDWI), and LULC Classification

Six remote sensing images acquired in the summer (Table 1, Serial Number = 4, 5, 6) were used to produce multi-temporal sets of LULC maps of the study area for 1990, 1995, 2000, 2005, 2010 and 2015, with the assistance of the auxiliary data (Table 1, Serial Number = 1, 2, 3) and using maximum likelihood classification in ENVI^TM^ 5.1 (Exelis Visual Information Solutions, Inc., Boulder, CO, USA) [59]. The detailed image processing techniques, including radiometric calibration, geometric correction, image classification, and accuracy assessment, were described previously [60]. The “Present Situation of Land Use Classification (GB/T221010-2007)” and the actual details of the study area were employed to extract the characteristics of the remote sensing data. Images of the study area were used to classify the LULC into six types: construction land, farmland, forest, grassland, water, and unused land (Table 2). Manual correction was then applied to ensure the accuracy of the classification. The accuracies of the classified products were assessed by manual interpretation using Google Earth Pro^®^. In total, 5% of the patches measuring more than 15 ha were selected randomly as samples. Using the spatial join function in ESRI ArcGIS^TM^ version 10.0 (ESRI, Redlands, CA, USA) [61], the manual interpretation results and the original results for these selected samples were compared to produce confusion matrixes [62]. The results of the Jeffries-Matusita distance separability inspections were all above 1.8000. The accuracies of the maps were above 90% in all 6 years. Finally, the kappa indices were calculated and the results were above 0.85 in all 6 years. These values satisfied the accuracy requirements for land-use change analysis [62]. All of the spatial data were transformed to a uniform coordinate system (datum: Beijing_54, ellipse: Krasovsky, projection: Transverse Mercator, zone: 19N).

The *NDVI* is a proxy of vegetation cover, which is frequently used in ecological and environmental studies:

(1) $$NDVI=(\rho_{NIR}-\rho_{RED})/\left(\rho_{NIR}+\rho_{RED}\right)$$

The *MNDWI* can enhance open water features while efficiently suppressing and even removing built-up land noise as well as vegetation and soil noise, and thus *MNDWI* is suitable for enhancing and extracting water information for a water region with a background dominated by built-up land areas [63]. We used *MNDWI* to represent water areas.

(2) $$MNDWI=(\rho_{Green}-\rho_{MIR})/(\rho_{Green}+\rho_{MIR})$$

*IBI* was proposed for the rapid extraction of construction land features in satellite imagery [64]:

(3) IBI=2ρMIR/(ρMIR+ρNIR)−[ρNIR/(ρNIR+ρRed)+ρGreen/(ρGreen+ρMIR)]2ρMIR/(ρMIR+ρNIR)+[ρNIR/(ρNIR+ρRed)+ρGreen/(ρGreen+ρMIR)]

where $\rho_{Green}$, ρRed, $\rho_{NIR}$, and $\rho_{MIR}$ represent the surface reflectivity of the green band, red band, near-infrared (NIR) band and, mid-infrared band, respectively, of the Landsat satellites images after Fast Line-of-sight Atmospheric Analysis of Hypercubes (FLAASH) atmospheric correction with ENVI 5.1.

#### 2.3.3. Retrieval of LST and Measurement of Relative SUHIs

##### LST Inversion

In this study, the LST was inverted from Landsat thermal infrared data (Table 1, Serial Number = 4, 5, 6, resampled to a spatial resolution of 30 m) in terms of TM-6 or ETM+-6 using the single-channel algorithm [65], and for TIRS-10/11 using the generalized single-channel method [66] combined with the split-window covariance-variance ratio technique (SWCVR) [67]. The main steps in this process comprised: (1) correcting the radiometric and geometrical distortions; (2) converting calibrated digital numbers (DNs) into absolute units of at-sensor spectral radiance; (3) converting the at-sensor spectral radiance into the at-sensor brightness temperature; and (4) correcting for the spectral emissivity of different land cover types (*NDVI* values) to generate the LST data [34,65,66,67]. More details of the computational process as follows:

Step 1: At-sensor radiance (*L_sen_*).

(4) Lsen=gain×DN+offst

Gain and offset are the slope and intercept of the radiance/digital number (DN) conversion function, respectively, which were obtained from the metadata files.

Step 2: At-sensor temperature (*T_sen_*).

(5) $$Tsen=K_{2}/\ln({K_{1}/L}_{sen}+1)$$

where the two correction constants *K*_1_ and *K*_2_, whose unite is in W/(m^2^·sr·μm) and *K*, respectively. More details value were shown in Table S1 (Supplementary Materials).

Step 3: Intermediate parameters.

(1) Parameter *γ* and *δ*

Refer to TM-6 and ETM+-6:

(6) $$\gamma =\frac{T_{sen}^{2}}{14387.7L_{sen}}{\left(\frac{\lambda^{4}}{1.19104\times 10^{8}}L_{sen}+\lambda^{-1}\right)}^{-1}$$

(7) δ=−γLsen+Tsen

Refer to the band 10 of Landsat 8:

(8) $$\gamma \approx \frac{T_{sen}^{2}}{b\gamma L_{sen}}$$

(9) $$\delta \approx T_{sen}-\frac{T_{sen}^{2}}{b\gamma }$$

Here, *b_γ_* = 1324.

(2) Atmospheric parameters (*ψ*_1_, *ψ*_2_, *ψ*_3_ and *w*)

(10) $$\begin{array}{c} \varphi_{1}=a_{1}\omega^{2}+b_{1}\omega +c_{1}\\ \varphi_{2}=a_{2}\omega^{2}+b_{2}\omega +c_{2}\\ \varphi_{3}=a_{3}\omega^{2}+b_{3}\omega +c_{3} \end{array}$$

The values of constants (*a*_1_, *a*_2_, *a*_3_, *b*_1_, *b*_2_*, b*_3_*, c*_1_, *c*_2_ and *c*_3_) were shown in Table S1. The *w* is water vapor content in g/cm^2^, which can be obtained from atmospheric profile (TM-6 and ETM+-6) or SWCVR method (TIRS-10):

(11) $$Fv={\left(\frac{NDVI-NDVI_{S}}{NDVI_{V}-NDVI_{S}}\right)}^{2}$$

where *FV* is the vegetation fraction, *NDVI_S_* and *NDVI_V_* correspond to the bare soil and fully-vegetated *NDVI*, respectively, which can be extracted from the *NDVI* histogram according to the cumulative percentage (5% sand 95% respectively) in the corresponding *NDVI* data.

Refer to TM-6 and ETM+-6:

(12) φ1=1τ,φ2=−Ldown−Lupτ,φ3=Ldown

*τ* is transmissivity; *L_up_* and *L_down_* are up-welling and down-welling atmospheric radiance, respectively, which were calculated using a web-based tool (http://atmcorr.gsfc.nasa.gov).

Refer to the band 10 of Landsat 8:

(13) $$R_{11,10}=\frac{\sum_{k=1}^{N}\left(T_{10,k}-{\bar{T}}_{10})(T_{11,k}-{\bar{T}}_{11}\right)}{\sum_{k=1}^{N}{\left(T_{10,k}-{\bar{T}}_{10}\right)}^{2}}$$

where *T*_10,*k*_ represents the brightness temperature of TIRS band 10 for pixel *k*; *T*_11,*k*_ represents the brightness temperature of TIRS band 11 for pixel *k*; ${\bar{T}}_{10}$ and ${\bar{T}}_{11}$ represents the mean brightness temperature of TIRS band 10 and 11 over N pixels, respectively.

(14) ε10ε11={0.9939NDVI<NDVIs0.9939NDVIv<NDVI(0.0195FV+0.9688)(0.0149FV+0.9747),NDVIs ≤NDVI≤NDVIv

(15) ω={−18.973(ε10ε11)R11,10+19.130,(ε10ε11)R11,10>0.9−13.412(ε10ε11)R11,10+14.158,(ε10ε11)R11,10<0.9

(3) Land surface emissivity (ε)

(16) $$\varepsilon =\{\begin{array}{cc} 0.97, & NDVI<NDVI_{s}\\ 0.97(1-FV)+0.99FV, & {\mathrm{NDVI}}_{s}\;\leq NDVI\leq \\ 0.99, & NDVI_{v}<NDVI \end{array}NDVI_{v}$$

Step 4: Land surface temperature (*LST*).

(17) $$Ts=\gamma [\varepsilon^{-1}(\psi_{1}L_{\mathrm{sn}en}+\psi_{2})+\psi_{3}]+\delta -273.15$$

##### Measurement of the Relative SUHIs

• Green Space Cooling Island Intensity

The *GSCI* intensity is calculated as:

(18) GSCI=ΔT=Tu−Tgs

where *T_gs_* is the daily average LST_m_ for a certain UGS interior and *T_u_* is the daily average land surface temperature measured by temperature and humidity probes (LST_m_) in the external 10 m buffer (excluding other green spaces and water) of the corresponding UGS, which is sufficiently wide to include the neighboring urban thermal information for roads, residential or business buildings, and parking spaces. In this context, the unit for temperature is degrees Celsius (°C). The thermal field variance index (LST grade/heat island intensity) was graded into five levels (Table 3). The spatial distribution of heat islands in the study area is shown in Figure 3.

• Thermal Landscape

The thermal landscape in the urban and suburban areas was divided into five categories using the LST mean-standard deviation method [68,69] (Table 3).

#### 2.3.4. Landscape Pattern Analysis

To assess the changes in the structural characteristics of the land and thermal landscapes at a scale of 100 m from 1990 to 2015 (5-year interval), the FRAGSTATS 4.2 program (University of Massachusetts, Boston, MA, USA) [70] was used to calculate the following six landscape metrics: fractal dimension index (FRAC), percentage of landscape (PLAND), aggregation index (AI), division index (DI), Shannon’s diversity index (SHDI), and expansion intensity (EI) (Table 4). These metrics have been used frequently to assess the structural characteristics of landscapes and to monitor changes in land use [44,53,71,72,73].

#### 2.3.5. Surveying and Measurement of SUHI-Related Indicators at the Plot Level

##### Size and Shape of UGSs

We used the Google Earth Professional online platform (Version 7.1) and images captured on 25 July 2015 under the same conditions (perspective elevation of ca. 1500 m and a parallel projection state). We delineated boundary polygons of 34 representative green spaces (area larger than 1 ha; Figure 2) with relatively even distributions in the urban core area, which ranged in size among large, medium, and small [75,76]. Each boundary polygon was saved separately in the form of a KML file, which was then transformed into a shape file (shp) in Global Mapper V10.01 and employed for extracting area, perimeter, external buffer, and subset/clipping-related spatial data. The UGS area, perimeter, and landscape shape index (*LSI*) were used to describe the UGS size and shape, and the perimeter/area ratio described the complexity and the edge effect for an urban park. A larger *LSI* indicated a more complex urban park shape [24]. *LSI* was calculated using ArcGIS 10.0 as follows:

(19) $$LSI=\frac{P_{t}}{2\sqrt{\pi \times A}}$$

where *P_t_* is the total perimeter around a green space and *A* is the area of the green space.

##### Surveys of UGS Forest Structure and Temperature Measurements

We determined the urban forest structures based on the tree-layer species number (TSN), TGB and LAI for forests in the UGSs according to our preliminary investigations. The number of plots sampled for each green space is shown in Figure 2. Each of the 34 sampling quadrats was defined as a 20 m × 20 m square (0.04 ha).

TSN: Number of species (diameter at breast height ≥5 cm, with a certain number) comprising the tree layer of a forest in an UGS.

LAI: One half the total leaf surface area per unit ground area [77]. Every tree in each sampling quadrat was measured directly with a canopy analyzer (fish-eye camera) and adjustable tripod. Each single tree’s LAI was calculated using analysis software (WinSCANOPY Reg v2005a) and the mean value was taken as the LAI for the whole sampling quadrat.

TGB: The space volume occupied by living plant stems and leaves [78]. TGB was evaluated for each tree in a sampling quadrat using an empirical formula according to corresponding shape of the tree canopy (Table 5).

The near-ground (approximately 1.5 m above the ground) air temperature (AT_1.5_) and land surface temperature (LST_m_) were measured using temperature and humidity probes (Yangling Qiantai Electronic Science and Technology Co. Ltd., Yangling, China) outside (30 m from the center, without trees) and inside 34 different types of green space during 26–31 July 2015, from 08:00 to 18:00, under roughly the same weather conditions (sunny, cloudless and wind-free). The data recording interval was 20 min and the daily average values were used in the statistical analyses.

#### 2.3.6. Statistical Analysis

Ordinary least squares multiple linear regression models and correlation coefficients were used to determine the effects of the UGSs configurations on LST_m_ at a scale of 100 m. In the analysis of the relationships between the linear regression models, GSCI/LST was used as the dependent variable, and the amount of urban vegetation (LAI and TGB) and shape (LSI) of the UGSs were used as independent variables. Spatial linear regression analysis of LST versus NDVI, FV, IBI and MNDWI in urban and suburban areas were performed by using SAS 9.2 (SAS Institute Inc., Cary, NC, USA) in order to analyze their quantitative relationships during the six study periods based on over 500 random sampling points with distance ≥200 m, which were evenly distributed in the study area.

## 3. Results

### 3.1. Relationship between UHIs and LUCC at the Regional Level

#### 3.1.1. Characteristics of the Mean Annual and Monthly Air Temperature, and Summer Heat Islands

**Figure 4:**
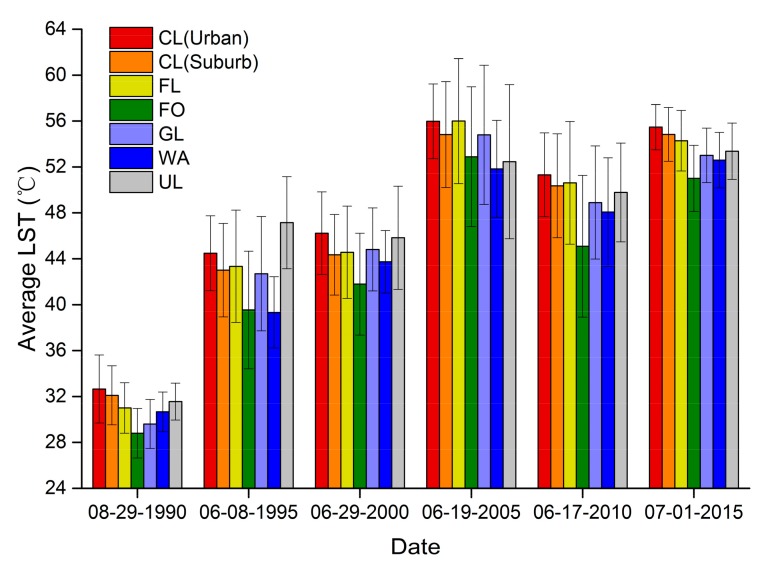
Comparison of the average LST among different LULC types and different regions (urban area of Yan’an city, and suburban area of Yan’an city) during six periods from 1990 to 2015 (error bars represent the standard deviation in the corresponding average LST).

June, July and August were the warmest months of the year (Figure 4) and we used Landsat satellite images, including the thermal infrared bands, for LST retrieval. According to the meteorological station at Yan’an (36°36′ N, 109°30′ E; altitude: 958.5 m), the average diurnal air temperature was 18.7 °C on 29 August 1990, 24.1 °C on 8 June 1995, 24.6 °C on 29 June 2000, 26.0 °C on 19 June 2005, 25.8 °C on 17 June 2010, and 27.5 °C on 1 July 2015. The high temperature in 2015 was attributable to the strong global EI Nino climatic effect as well as severe local deforestation and construction activities due to construction of the new Yan’an city area, which caused dry and warm weather. These effects are readily discernible in the annual mean air temperature charts shown in Figure S1.

Figure S2 showed the 20-min variations in the SUHI intensity at 34 open impervious surface sites located close to the centre of Yan’an City in July. The SUHI intensity values ranged from −6.9 °C to 10.1 °C, and the standard deviation ranged from −2.7 °C to 9.4 °C during the day (08:00–18:00), where the SUHI occurred predominantly during the nearly noon-time hours and it reached a maximum by 11:40 (Beijing Time).

#### 3.1.2. Variations in the LST among Different Land Use Types

The average LST distributions during the six research periods for urban area of Yan’an city (UA), and suburban area of Yan’an City (SUA) are showed in Figure 4. In the two regions (UA and SUA), the lowest average LST always occurred in forestland and water area among the six LULC types during the six research periods. By contrast, the highest average LST occurred in construction land (1990, 2000, 2005, 2010 and 2015) or unused land (1995) because of the bare soil in those two LULC types caused by soil erosion, which mainly affected by local anthropogenic activities, such as land reclamation, oil exploitation, and urbanization. A comparison of the average LST among the two regions (UA and SUA) showed that the average LST for each LULC type in the descending order by UA and SUA. Thus, these were significant spatial gradients in the LST from the city center to the surrounding suburban area. In Figure 4, the length of the error bar represents the spatial difference in the LST, where a longer error bar indicates the higher heterogeneity of the corresponding thermal landscape between the two regions (UA and SUA) for each LULC type. In general, the length of the error bar for each LULC type in the ascending order by UA and SUA, from 1995 to 2015, which also suggested that the heterogeneity and fluctuations in the average LST decreased gradually along a suburban-urban gradient in Yan’an City.

#### 3.1.3. Relationships between the Spatial Distributions of SUHI and LULC

**Figure 5:**
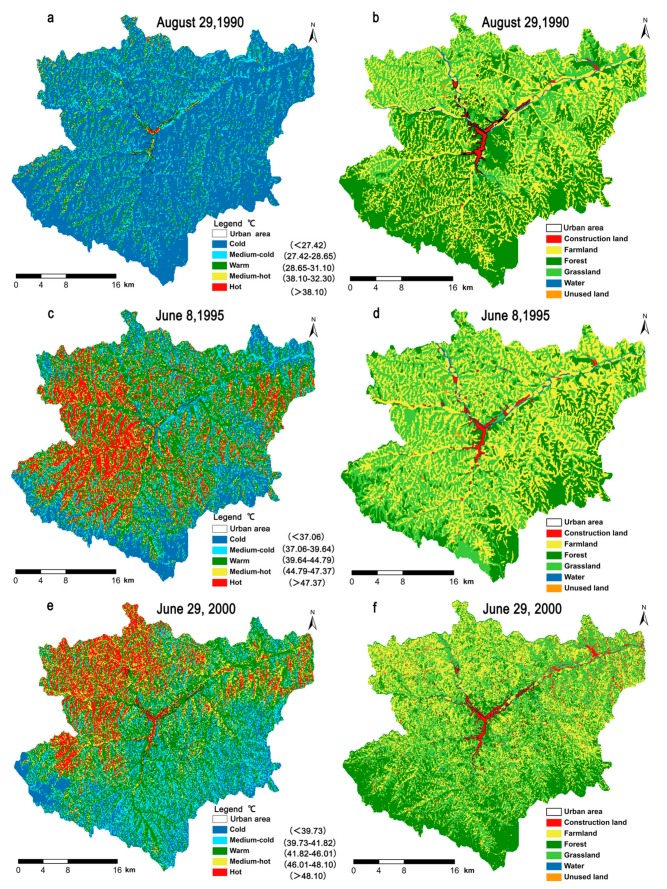

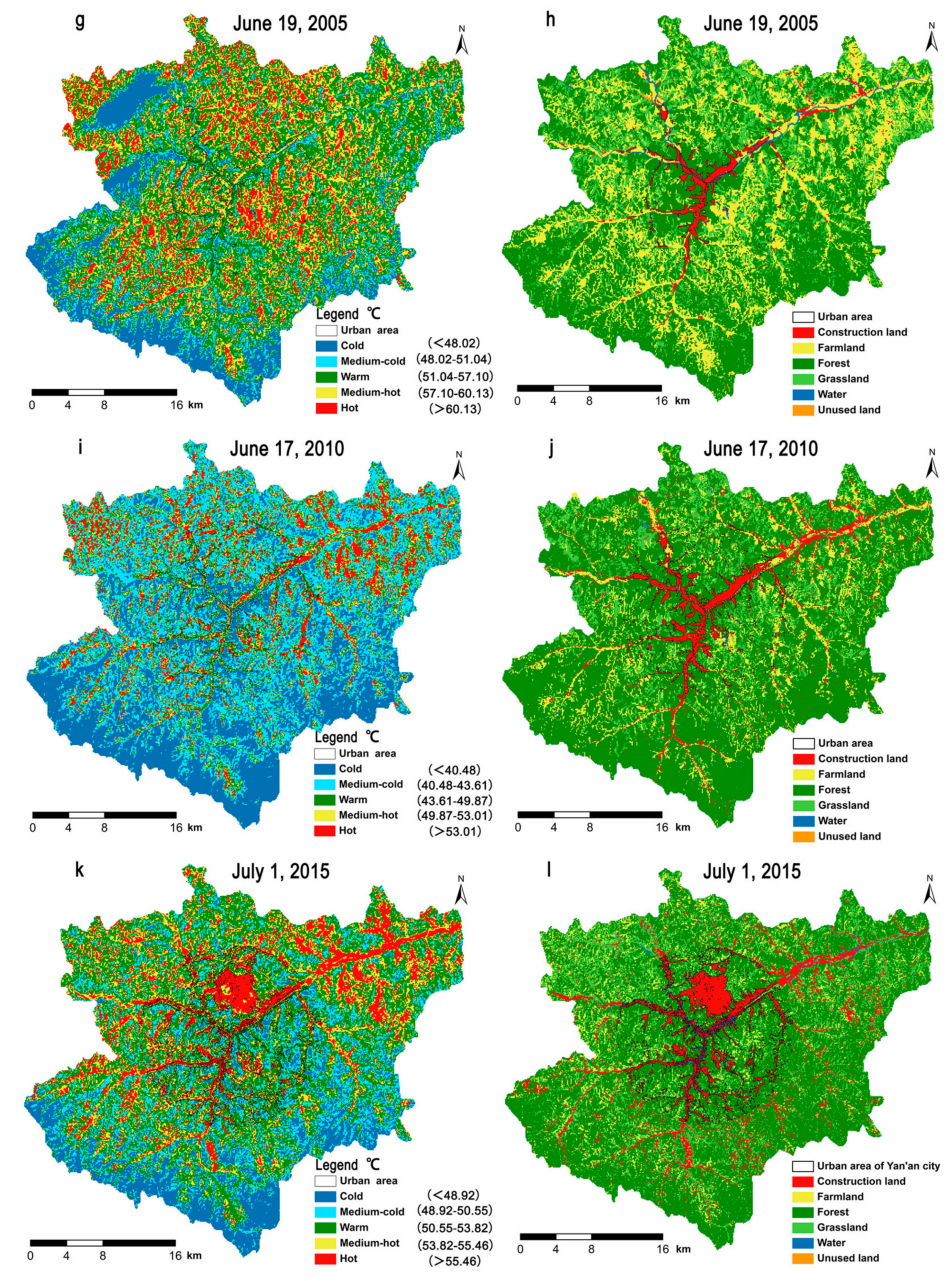
(**a**–**f**) Maps comparing the spatial distribution of the land surface temperature and land use/cover during the three periods from 1990 to 2000; (**g**–**l**) Maps comparing the spatial distribution of the land surface temperature and land use/cover during the three periods from 2005 to 2015.

In this study, we selected six Landsat images (Table 1), where the distribution of urbanized areas and SUHIs (medium-hot and hot areas) changed from a sparsely dotted pattern in 1990 to a chain/areal pattern in 2015 due to the gradual increase in the urban area of Yan’an city during the study period. A comparison of the LULC maps derived from images acquired on 29 August 1990 and 1 July 2015 shows the dramatic expansion of the urbanized area by 2015 (Figure 5). In 1990, the hot areas only occurred on construction land at the center of Yan’an city (Figure 5a,b). In 1995 and 2000, the hot areas were clumped on construction land, farmland, and grassland (Figure 5c–f). In 2005 and 2010, the hot areas were scattered on construction land and farmland (Figure 5g–l). In 2015, the hot areas were mainly distributed on construction land (Figure 5k–l).

According to the spatial-temporal analysis of the variations in the SUHI intensity at 5-year intervals, construction land had the highest LST. There were significant spatial gradients in the LST from the city center to the surrounding suburban and rural areas.

Based on land use/cover changes trajectory tracking, from 1990 to 2015, the amount of construction land in the urban area increased by 56.72 km^2^, from 11.53 km^2^ to 68.25 km^2^, where 33.32 km^2^ of UGS was transformed into construction land. Therefore, 42.42% (17.61 km^2^) of the new land use types (41.52 km^2^ in all) were transformed from UGS. Table S2 shows the time series of the six types of land use/cover area in percentage.

#### 3.1.4. Relationship between SUHI and LULC

**Figure 6:**
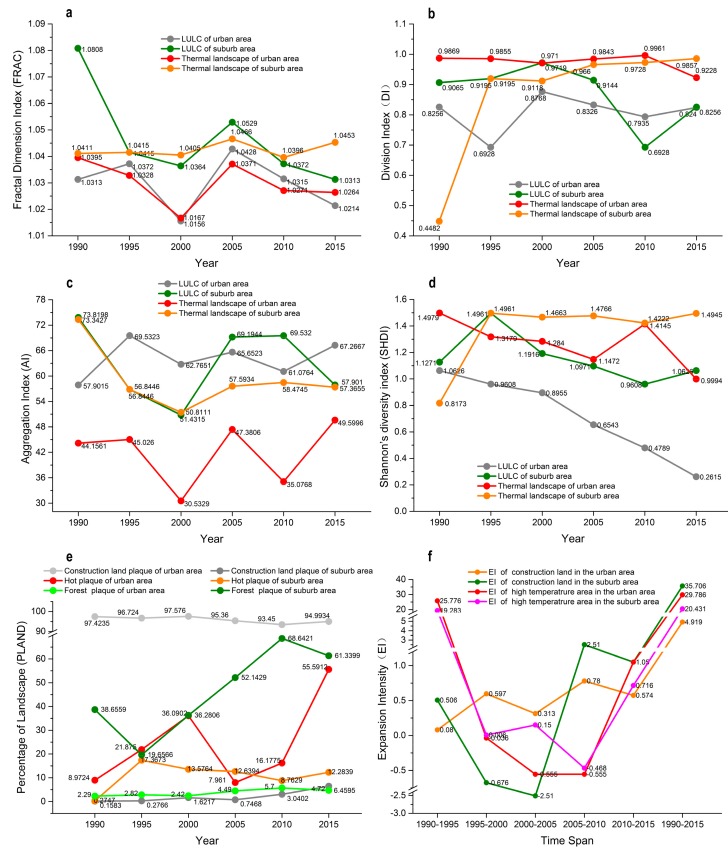
Changes in the landscape metrics for the LULC and thermal landscape in urban and suburb areas: (**a**) fractal dimension index (FRAC); (**b**) division index (DI); (**c**) aggregation index (AI); (**d**) Shannon’s diversity index (SHDI); (**e**) percentage of landscape (PLAND); and (**f**) expansion intensity (EI).

At the landscape scale, the spatiotemporal changes in the six landscape metrics (FRAC, DI, AI, SHDI, PLAND and EI) for the thermal landscape (classified LST) were similar to those in the corresponding urbanization landscape metrics for LUCC (especially construction land) during the last 25 years. The landscape metrics showed that long-term anthropic intervention (petroleum exploitation and rapid urbanization) produced a highly fragmented and diversified landscape, as shown by the decrease in FRAC for LULC and thermal landscape because of the decline in the natural landscape (Figure 6). The decreases in DI of LULC and thermal landscape of urban area, except for thermal landscape of suburb area (Figure 6b), and the increases in AI, PLAND and EI for urban area (construction land) because of urbanization (Figure 6c,e,f). The decrease in SHIDI (Figure 6d) showed that there was a less dispersed and even distribution of land-use types, especially in the new Yan’an city, which was confirmed by the landscape spatial distribution analysis (Figure 5). 

Under the macroscopic land use policy, several large-scale land surface transformation activities have occurred, where petroleum exploitation (initiated in 1990s), the “Grain-to-Green” program (initiated in 1999), and the leveling of mountains to build cities (initiated in 2012) were the most influential projects, which are responsible for the turning points in 2000, 2005 and 2010 in the broken line graphs for the six landscape metrics in Figure 6.

### 3.2. Effects of UGS Size, Shape, and Tree-Layer Structures on GSCI

Table 6 shows the descriptive statistics for GSCI, and the size and shape of UGSs. The mean value and standard error (±SE) for LSI, LAI, TGB, and GSCI were 1095.5 ± 1216.9 m^2^, 1.421 ± 0.395, 2.252 ± 0.897, 12852.3 ± 2814.0 m^3^, and 3.66 ± 1.78 °C respectively. 

The top ten GSCI values (5.10 °C to 8.57 °C, GSCI order, 1–10) were all observed in the larger green spaces comprising coniferous broadleaf mixed, deciduous broadleaf mixed, or larger tree canopy pure forests. The tree species compositions of the top 10 spaces in terms of the cooling effect were (see the definitions of the abbreviations in Table 6): (1) 6PT + 4RP + 3PO + 3SJ + 3PCa + 2SC + 2PH + 1PA, (2) 20PB + 36SJ + 7SM + 4As + 2GB + 1PH + 1PT, (3) 15GB + 5UP + 3JF, (4) 27PH, (5) 100RP, (6) 7SM + 5PT + 3PA + 2SJ + 1JF, (7) 5ZJ + 3AV + 3PU, (8) 34SM + 7GB + 5PT + 5JF + 4SJ, (9) 5PA + 4SM + 2PT + 1SJ + 1GB, and (10) 6PCa + 4SJ + 3SM + 2PH.

Based on GSCI order, after considering the diversity of tree species and the cooling effect, the top three tree species compositions for UGSs in the urban area of Yan’an city were: (1) 6PT + 4RP + 3PO + 3SJ + 3PCa + 2SC + 2PH + 1PA (2) 20PB + 36SJ + 7SM + 4As + 2GB + 1PH + 1PT, and (3) 15GB + 5UP + 3JF.At the sampling plot scale, the linear regression analysis based on ordinary least squares showed that there were significant strong positive relationships between GSCI and LAI, and the logarithm of TGB, and there was a negative correlation (*p* < 0.05) between GSCI and LSI when LSI between 1.062 and 1.717, while GSCI_LSTm_ had a significant positive correlation with GSCI_AT1.5_ according to their corresponding independent variables (Table 7).

### 3.3. Temporal-Dynamic Linear Correlation between the Remote Sensing Ground Indexes and LST

**Figure 7:**
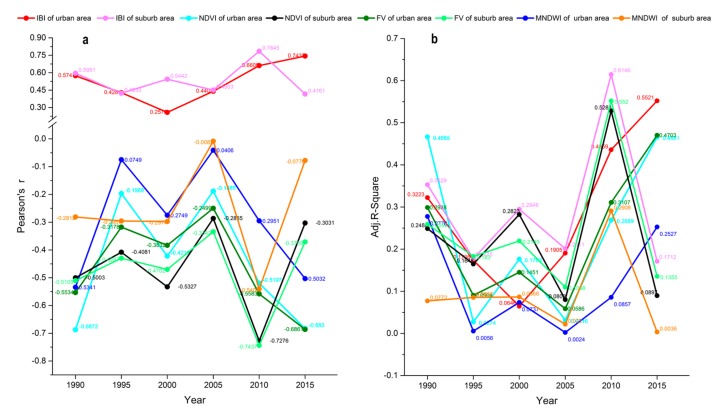
Linear correlation analysis of LST versus the remote sensing ground indexes (NDVI, FV, IBI and MNDWI) in urban and suburban areas: (**a**) Pearson’s correlation coefficients (r); (**b**) adjusted R-square values (R^2^_adj_) of spatial linear regression.

Figure 7 shows the following results: (1) There was a significant strong positive correlation between LST and IBI, and inverse correlations between LST and FV, NDVI, and MNDWI in both the urban and suburban areas during the six periods. (2) The correlation coefficients (Pearson’s r) differed for the ground remote sensing indexes (FV, NDVI, IBI, MNDWI) versus LST, where in urban areas, IBI was the key positive factor related to the increases in LST, whereas NDVI and MNDWI were the crucial factors related to decreases in LST during the six periods. 

The absolute average values of the correlation coefficients during the study period were ranked as follows: for the urban area, IBI (0.5174) > FV (−0.4581) > NDVI (−0.4495) > MNDWI (−0.2871); and for the suburban area, IBI (0.5356) > FV (−0.4765) > NDVI (−0.4597) > MNDWI (−0.2501). In general, the average Pearson’s r and adjusted R-square have shown that the correlations between the four remote sensing ground indexes and LST were stronger in the suburban area than the urban area. (3) On the five-year temporal scale, the changes in Pearson’s correlation coefficients (r) and adjusted R-square values (R^2^_adj_) of spatial linear regression emerged four turning points (1995, 2000, 2005 and 2010), the similar to the trends of FRAC in Figure 6a, because of significant changes of land use/cover every five years from 1995 to 2010.

## 4. Discussion

### 4.1. Main Reasons for the Changes in Vegetation and LST

In response to the national “Grain-to-Green” program, Yan’an Municipal People’s Government issued the “Closing the mountain and planting trees, feeding animals in shed” act on 16 October 1999. Subsequently, deforestation was replaced by afforestation. Moreover, the establishment of Yan’an city as a “National Forest City” was affirmed by the State Forestry Bureau of China on 29 January 2012. Yan’an Municipal People’s Government initiated the “National Forest City” establishment project and issued related operational documents, such as “The overall planning of construction for national forest city in Yan’an” and “Overall planning of Yan’an City (2012–2030).” However, specific weather conditions and large-scale development projects affected the urban forest and its construction. For example, torrential rain occurred during 2013, where the total precipitation in 17 days (from 28 May to 7 October) was 725 mm, while the annual precipitation was 929.1 mm, which was nearly twice of the normal year. Petroleum exploitation and rapid urbanization, including the leveling of mountains to build the new city, meant that all the vegetation was removed from over 30 hills, i.e., a total area of 78.5 km^2^. The changes in vegetation were significantly influenced by these land use policies and human activities. In the suburban area, due to ecological restoration, the average vegetation cover (forest land) increased by 56.54% (from 34,172 ha to 53,493 ha) between 1990 and 2015, which was similar to the trend in Baota District [66]. By contrast, in the urban area, the average vegetation cover (forest land) decreased by 11.15% (from 17,661 ha to 15,692 ha) between 1990 and 2015. The leveling of mountains to build the new city area was the major cause for the decrease in the urban vegetation coverage and the corresponding increase in the LST [56].

### 4.2. Correlations between Different Land Surface Indicators and LST

Odindi et al. [33] noted that impervious surfaces are heat sources, whereas green spaces are the major heat sinks in Ethekwini Municipal Area, South Africa. Impervious surfaces and bare land are high temperature zones [31,74], and construction land, farmland, and unused land contained high proportions of impervious surface and bare land in our study area. Our results showed that construction land, farmland and unused land had higher average LST values than the other types of land use during the six study periods (Figure 4). Our results also showed that the four ground indexes were all significantly correlated with the mean LST, i.e., a positive correlation with IBI and negative correlations with FV (vegetation fraction), NDVI (normalized difference vegetation index) and MNDWI (modified normalized difference water index), shown in Figure 7. These findings are consistent with those of other studies. For example, Xu [64] found a significant positive relationship between the average LST (land surface temperature) and IBI (an index-based built-up index), while other studies reported significant negative relationships between the mean LST and the amount of impervious surface [79,80], NDVI [43,51,81] and MNDWI [64]. Related studies had shown that the key factors affecting urban LST are not only land cover patterns, but also other anthropogenic forces, especially land use. Therefore, the explanation of urban LST or SHUI (surface urban heat island) by land cover alone is inadequate. Especially at fine spatial scales, information on land use is more meaningful than that of land cover to indicate the impacts of urbanization on ecosystems [3,5,36,41,47,51,52,81].

### 4.3. Spatial Characteristics of LULC and Variations in Vegetation vs. LST Along the Urban-Rural Gradient

We found that the zones closest to the city center did not have the highest average LST values in all six periods (Figure 5). Considering that the percentage of construction land and forest (Table S2) had a strong significant correlation with the mean LST, then PLAND (percentage of landscape)for construction land and forest helped to explain why the zones close to the city center did not always have the highest mean LST (Figure 5c). The changes in the spatio-temporal distribution of the high LST zone were clearly divided into two stages: in the first stage from 1990 to 2005, the high LST zones had a plaque-like distribution in farmland (Figure 5a–h); and in the second stage from 2010 to 2015, the high LST zones were distributed mainly in construction land (Figure 5i–l). Previous studies have shown that some screened landscape pattern metrics can be used to delineate the effects of uraban heat islands (UHIs) [44,70,72,82]. In the present study, six urban landscape evaluation indexes. i.e., FRAC (fractal dimension index), PLAND, AI (aggregation index), DI (division index), SHDI (Shannon’s diversity index) and EI (expansion intensity) and previous research conclusions were employed to calculate the thermal and land-type characteristics of the two regions (urban area and suburban area) in the study area. The PLAND indicator showed that the area of impervious surface (construction land) and land surface temperature increased in the study area. The EI indicator demonstrated that the intensity of the increase in the construction land and land surface temperature were greater during 2005–2015. The FRAC indicator showed that the regional complexity of LST and LULC were decreasing, and revealed that the reduction in patch boundary complexity and degeneration of natural landscape. The AI and DI indicators demonstrated that the spatial division and aggregation of the different LST grades changed consistent with that of the land use/cover through the study periods (Figure 6). These findings highlight the relationship between the LULC pattern and LST at the 100 m landscape scale.

### 4.4. Major Factors That Influenced the GSCI Intensity

Our results demonstrate that the shape of UGSs as well as features of the urban forest structure (TSN, LAI and TGB) in UGSs significantly affected the magnitude of the GSCI (green space cooling island) intensity (Table 6 and Table 7). We found significant positive relationships between GSCI and TSN, LAI and GSCI_AT1.5_. Ren et al. [24] also found that the park cooling island intensity had significant positive correlations with features of the urban forest structure in parks (i.e., canopy density, LAI, basal area, height, diameter and stem density), while Vidrih and Medved [83] showed that the optimal length for a park with LAIsp = 3 (specific dimensionless coefficient of the leaf area) to achieve the best cooling intensity is 130 m. Tree volume had the highest impact on the nocturnal UHI intensity in Amsterdam within 40 m and a one degree reduction in temperature was predicted for an increase in the tree canopy volume of 60,000 m^3^ in its 40 m buffer [84].

## 5. Conclusions

Urban heat islands (UHIs), especially surface urban heat islands (SUHIs), are mainly influenced by rapid local urbanization. The spatial variations in the LST levels in Yan’an City were related to SUHIs and LUCC (land use and cover change) from 1990 to 2015 with five-year intervals were revealed in this studies. The higher LST values were usually distributed in low vegetation cover land types (construction land, farmland and unused land). Land surface remote sensing indexes, an index-based built-up index (IBI), normalized difference vegetation index (NDVI), vegetation fraction (FV) and modified normalized difference water index (MNDWI) and landscape pattern metrics, i.e., Fractal dimension index (FRAC), percentage of landscape (PLAND), aggregation index (AI), division index (DI), Shannon’s diversity index (SHDI) and expansion intensity (EI) described the spatiotemporal relationships well between SUHI and LUCC at the landscape scale (100 m) in our study area. Urban green spaces (UGSs) can mitigate SUHIs at the sample scale (20 × 20 m) and reduce the LST by 0.67 °C to 8.57 °C. Our results demonstrated that several factors affected the GSCI (green space cooling island) intensity, i.e., positive effects of the tree canopy (LAI) and TSN (number of species comprising the tree layer of a forest in an UGS, diameter at breast height above 5 cm, with a certain number), a negative effect of UGS shape (LSI), logarithmic positive effects of the spatial green mass of trees and shrubs, i.e., the space volume occupied by living plant stems and leaves (TGB), and complex relationships with the configuration of tree layer species. We found that mixed forest was better than that pure forest for mitigating SUHIs. According to the important value (VI) of tree species in the 34 sample plots (Table S3), the increased use of native plants with higher VI (i.e., dominant species), such as ginkgo (*Ginkgo biloba* L.), lacebark pine (*Pinus bungeana* Zucc. ex Endl.), Hebei poplar (*Populus hopeiensis* Hu et Chow), Chinese pine (*Pinus tabuliformis* Carr.), *Robinia* (*R. pseudoacacia* Linn.), Chinese scholar tree (*Sophora japonica* Linn.), willow (*Salix matsudana* var. *matsudana* f. *pendula* Schneid.), and elm (*Ulmus pumila* L.), may be a suitable precautionary measure for urban greening in Yan’an City and other regions with similar climates.

## Figures and Tables

**Table 1:** Overview of the data used in this study.

Serial Number	Data	Acquisition Date and Time (GMT)	Spatial Resolution	Utility
1	SPOT-5	9 September 2003; 03:40:49 9 September 2003; 03:40:57	2.5 m 2.5 m	Land use/cover classification of satellite imagery
2	GeoEye-1	2009	1.65 m	
3	Land use map	1995, 2000	1:100,000	
		2011	1:10,000	
4	Landsat 5 TM	29 August 1990; 02:39:18 8 June 1995; 02:25:55 19 June 2005; 03:06:59 17 June 2010; 03:10:03	30 m, 120 m	Used for land use/cover type classification, remote sensing and index calculation. Thermal infrared bands used for retrieving land surface temperature values.
5	Landsat 7 ETM+	29 June 2000; 03:11:06	30 m, 60 m	
6	Landsat 8 OLI & TIRS	1 July 2015; 03:18:49	30 m, 100 m	
7	Boundary map of Yan’an city area	2011		Subset related data.

**Table 2:** Land use/land cover type classification system used in this study.

Primary Types	Abbreviation	Secondary Types	Code
Construction land	CL	Urban area, rural residential area, other construction land	1
Farmland	FL	Paddy field, non-paddy field	2
Forest	FO	Forest, shrubs, sparse forest, other forest	3
Grassland	GL	Dense grassland, moderately dense grassland, sparse grassland	4
Water	WA	River, lake, reservoir or pond, beach, bottomland	5
Unused land	UL	Sandy land, saline land, marsh, bare land, bare rock, other unused land	6

**Table 3:** Thermal landscape classification obtained using the mean-standard deviation method.

Thermal Landscape Category (LST Grade/Heat Island Intensity)	LST Division
Hot/extremely strong	T(*x*, *y*) ≥ *m* + *std*
Medium-hot/very strong	*m* + *std* > T(*x*, *y*) ≥ *m* + 0.5*std*
Warm/moderate	*m* + 0.5*std* > T(*x*, *y*) ≥ *m* − 0.5*std*
Medium-cold/weak	*m* − 0.5*std* > T(*x*, *y*) ≥ *m* − *std*
Cold/none	T(*x*, *y*) < *m* – *std*

T(*x*, *y*) is the surface temperature at location (*x*, *y*), and *m* and *std* are the mean and standard deviation for the LSTs, respectively.

**Table 4:** Evaluation indices for LST and LUCC types [72,73].

Evaluation Index	Description	Formula
Fractal dimension index (FRAC)	Ranging between 1 and 2, where a greater value indicates more complex characteristic of the plaque and landscape. *A* is the total area and *P* is the perimeter of a patch.	$FRAC=\frac{2\ln(0.25P)}{\ln A}$
Percentage of landscape (PLAND)	Characteristic of a certain class area relative to the proportion of the total. *A* is the total area, *a* is the plaque area, and *n* is the number of patches.	$PLAND=\frac{\sum_{i=1}^{n}a_{i}}{A}*100$
Aggregation index (AI)	Characterization of the degree of plaque accumulation, ranging between 0 and 100, where a lower value indicates a greater degree of dispersion for the representative. *g_ii_* is adjacent to a number of patches relative to a class plaque.	$AI=\left|\frac{g_{ij}}{\max\to g_{ij}}\right|$
Division index (DI)	Measure of the plaque distribution, ranging between 0 and 1, where a value closer to 1 represents a more severe split. *A* is the total area, *a_i_* is the area of the *i*th plaque, and *n* is the number of patches.	$DI=\left[1-\sum_{1}^{n}\left(\frac{a_{i}}{A}\right)\right]$
Shannon’s diversity index (SHDI)	Diversity measure that increases with the number of patch types and as the proportional distribution of the area among patch types becomes more equal.	$\mathrm{SHDI}=-\sum_{i=1}^{m}\left(Pi\times \ln Pi\right)$
Expansion intensity (EI)	Measure of the intensity of spatial expansion. *A_i + j_* and *A_i_* are the areas in years *i + j* and *i*, respectively.	$EI=\frac{A_{i+j}-A_{i}}{A_{i}}$

FRAC, PLAND, AI, DI and EI were calculated for LULC and SHUI in the whole area, suburban area and urban area at the landscape level [74].

**Table 5:** Empirical formulae for determining the three-dimensional green biomass of trees [78].

Canopy Shape	Cylinder	Oval	Sphere	Flat Spheroid	Cone	Spherical Fan	Spherical Segment
Empirical formula	$\frac{\pi x^{2}y}{4}$	$\frac{\pi x^{2}y}{6}$	$\frac{\pi x^{2}y}{6}$	$\frac{\pi x^{2}y}{6}$	$\frac{\pi x^{2}y}{12}$	$\frac{\pi \left(2y^{3}-y^{2}\sqrt{4y^{2}-x^{2}}\right)}{3}$	$\frac{\pi \left(3xy^{2}-2y^{3}\right)}{6}$
Tree species	PC, PH, PO, SC, WS	FC, PCa, SM, UP	AV, JF	AM, JR, PU, SJ	CD, GB, MA, PA, PB, PT, RP, ZJ	AP, FS, PS, SJv	AJ, SJp

*x*: tree canopy diameter; *y*: crown length; AJ: *Albizia julibrissin* Durazz; AM: *Acer mono* Maxim; AP: *Amygdalus persica* L.; AV: *Armeniaca vulgaris* Lam*.*; CD: *Cedrus deodara* (Roxb.) G. Don; FC: *Fraxinus chinensis* Roxb.; FS: *Forsythia suspensa* (Thunb.) Vahl f. *suspensa*; GB: *Ginkgo biloba* L.; JF: *Juniperus formosana* Hayata; JR: *Juglans regia* L.; MA: *Morus alba* L.; PA: *Picea asperata* Mast.; PB: *Pinus bungeana* Zucc. ex Endl.; PC: *Pistacia chinensis* Bunge; PCa: *Prunus cerasifera* Ehrhar f. *atropurpurea* (Jacq.) Rehd.; PH: *Populus hopeiensis* Hu et Chow in Bull.; PO: *Platycladus orientalis* (L.) Franco; PS: *Pinus sylvestris* Linn. var. *mongolica* Litv.; PT: *Pinus tabuliformis* Carr.; PU: *Pyrus ussuriensis* Maxim.; RP: *Robinia pseudoacacia* Linn.; SC: *Sabina chinensis* (L.) Ant.; SJ: *Sophora japonica* Linn.; SJp: *Sophora japonica* Linn. var. *japonica* f. *pendula* Hort.; SJv: *Sophora japonica* Linn. var. *violacea* Carr.; SM: *Salix matsudana* var. *matsudana* f. *tortuosa* (Vilm.) Rehd.; UP: *Ulmus pumila* L.; WS: *Wisteria sinensis* (Sims) Sweet.; ZJ: *Ziziphus jujuba* Mill.

**Table 6:** Descriptive statistics for the size, GSCI and number of plots for each urban green space.

Green Space	Sample Plot Code	Tree Species Composition	TSN	LSI	LAI	TGB (m^3^)	GSCI (°C)	GSCI Order
Zaoyuan revolution site	1	3AV + 2PU + 2JR	3	1.062	1.44	3598.3	2.67	25
	2	5ZJ + 3AV + 3PU	3	1.145	1.973	4838.1	4.98	7
	3	15GB + 5UP + 3JF	3	1.117	1.081	5952.6	5.54	3
	4	13ZJ + 8SC + 8RP + 3PC + 3MA	5	1.118	1.182	5110.8	4.83	11
Xibeichuan park	5	8PS + 6SM + 5FC + 7AM	4	1.247	1.379	3596	3.21	21
	6	31PT	1	1.327	1.030	3472.5	2.36	26
	7	100PS	1	1.399	2.060	2188.1	2.06	27
	8	14 SM	1	1.865	2.470	10,054.4	3.11	22
	9	10FC + 16PT	2	1.246	2.760	6329.6	3.98	16
	10	4PH + 2PT + 3PB + 1AV	4	1.228	1.930	8967.1	1.87	28
	11	63PO	1	2.278	0.980	23.7	0.67	34
	12	5SJ + 1AM	2	1.281	1.372	1088.5	1.41	30
	13	18PA	1	1.268	1.179	343.2	0.93	32
	14	1RP + 5PCa + 4PT + 1SM	4	1.133	1.459	2176.81	1.49	29
	15	64GB + 10UP	2	2.774	1.297	1120.3	1.26	31
Yan’an airport green space	16	7SM + 5PT + 3PA + 2SJ + 1JF	5	1.2	3.56	42,722.4	5.43	6
	17	6PCa + 4SJ + 3SM + 2PH	4	1.134	2.93	34073.2	4.86	10
	18	5PA + 4SM + 2PT + 1SJ + 1GB	5	1.202	3.24	8050.7	4.93	9
Yuying park	19	6PT + 4RP + 3PO + 3SJ + 3PCa + 2SC + 2PH + 1PA	8	2.007	4.567	154,618	8.57	1
Liulin green belt	20	20PB + 36SJ + 7SM + 4As + 2GB + 1PH + 1PT	7	1.217	3.755	56,100.4	6.39	2
Dalitang green space	21	4PCa + 3SJp + 2PT + 1AP + 1SC + 1PS + 1CD	7	1.385	2.859	5485.7	4.16	13
Shilipu nursery	22	100RP	1	1.25	3.140	12,403.3	5.44	5
	23	97JF	1	1.717	1.072	87.9	0.82	33
	24	27PH	1	1.807	2.980	15,246.6	5.51	4
Revolutionary memorial hall green space	25	34SM + 7GB + 5PT + 5JF + 4SJ	5	1.098	2.804	5397.1	4.95	8
	26	13SM + 13SJ + 3JF + 3PT	4	2.211	2.651	2065.7	4.76	12
	27	10GB + 10JF + 3SM	3	1.286	2.202	2964.1	4.06	14
Wangjiaping peach park	28	53AP + 1JR	2	1.217	1.830	2236	3.66	18
	29	3AV	1	1.458	2.583	2753.3	3.29	20
Yan’an university campus	30	11PO + 7SJv + 6SJp + 1AJ	4	1.377	2.628	1503.1	3.32	19
	31	19JR + 11PCa + 6JF + 4SJ + 4PA + 3AJ	6	1.249	2.307	28,408.4	3.78	17
	32	60WS	1	1.284	2.941	649.5	2.96	24
	33	22SJp + 19FS + 16PA + 16AP	4	1.575	2.786	2439.8	3.04	23
	34	41PO + 36JF + 24PCa + 4PA + 4PO	5	1.137	2.138	913.1	3.99	15
Mean				1.421	2.252	12,852.3	3.66	
Standard deviation				0.395	0.897	2814.0	1.78	

**Table 7:** Linear regression models for GSCI versus LAI, LSI, TGB, and AT_1.5_.

y	x	Model	Domain of Definition	R^2^	*p*-Value
GSCI	TSN	y = 1.99684 + 0.51597x	1 ≤ x ≤ 8	0.3248	0.0003
GSCI	LAI	y = 0.34095 + 1.4719x	0.980 ≤ x ≤4.567	0.5363	<0.0001
GSCI	LSI	y = 9.4506 − 4.644x	1.062 ≤ x ≤ 1.717	0.1760	0.0151
GSCI	LSI	y = 14.2984 − 4.7837x	1.717< x ≤ 2.774	0.1669	0.2307
GSCI	TGB	y = –2.9910 + 0.8089lnx	23.7 ≤ x ≤ 154618	0.6051	<0.0001
GSCI_LSTm_	GSCI_AT1.5_	y = 0.5252 + 0.2172x	0.67 ≤ x ≤ 8.57	0.4253	<0.0001

## Supplementary Materials:

The following are available online at www.mdpi.com/1660-4601/14/8/840/s1, Table S1: The constants for LST retrieval with different types of thermal infrared data, Table S2: The time series of the area percentage of the six land use/cover (%), Table S3: The importance value of tree species in the 34 sample plots of the UGSs (%), Figure S1: Fluctuations in the monthly (a) and yearly (b) mean air temperature from 1990 to 2015, where the mean value was calculated using data obtained from Yan’an city weather stations, Figure S2: The 20-min variations in the land surface heat island intensity during July 2015 under sunny weather at 34 open impervious surface sites located close to various green spaces in the core urban area of Yan’an City. The mean (blue line) and standard deviation (gray line) for each 20 min period are also shown.
