# Supplementary material for: Effects of Land Use/Cover Changes and Urban Forest Configuration on Urban Heat Islands in a Loess Hilly Region: Case Study Based on Yan’an City, China
Source: Int J Environ Res Public Health. 2017 Jul 26;14(8):840. doi: 10.3390/ijerph14080840 (PMC5580544; doi:10.3390/ijerph14080840)
Supplement: Supplementary file 1 [file ijerph-14-00840-s001.pdf]

Supplementary Materials

Table S1: The constants for LST retrieval with different types of thermal infrared data.

**Constants**	**Landsat 5 TM-6**	**Landsat 7 ETM+-61/62**	**Landsat 8 TIRS-10/11**
K_1	607.76	666.09	774.8853
K_2	1260.56	1282.71	1321.0789
λ (μm)	11.457	11.27	10.90
a_1	0.14714	0.14714	0.04019
a_2	-1.1836	-1.1836	-0.38333
a3	-0.04554	-0.04554	-0.00918
b_1	-0.015583	-0.015583	0.02916
b_2	-0.37607	-0.37607	-1.50294
b_3	1.8719	1.8719	1.36072
c_1	1.1234	1.1234	1.01523
c_2	-0.52894	-0.52894	0.20324
c_3	-0.39071	-0.39071	-0.27514

Table S2: The time series of the area percentage of the six land use/cover (%).

Year	Construction land		Farmland		Forest		Grassland		Water		Unused land	
	UA	SUA	UA	SUA	UA	SUA	UA	SUA	UA	SUA	UA	SUA
1990	3.67	0.21	39.16	38.51	34.89	39.18	20.53	20.66	1.47	0.72	0.29	0.72
1995	3.70	0.31	46.24	41.61	9.32	23.05	39.21	34.45	1.08	0.53	0.46	0.05
2000	5.91	3.27	35.18	30.57	17.26	31.00	40.32	34.53	1.33	0.64	0.00	0.01
2005	7.75	1.06	26.19	26.17	43.73	52.81	20.71	19.15	1.47	0.71	0.15	0.11
2010	13.80	3.71	11.95	12.67	56.16	69.98	16.60	12.82	0.64	0.36	0.85	0.46
2015	21.73	7.63	21.09	7.66	34.89	61.74	20.53	22.49	1.47	0.44	0.29	0.05

UA, Urban core area; SUA, Suburb area.

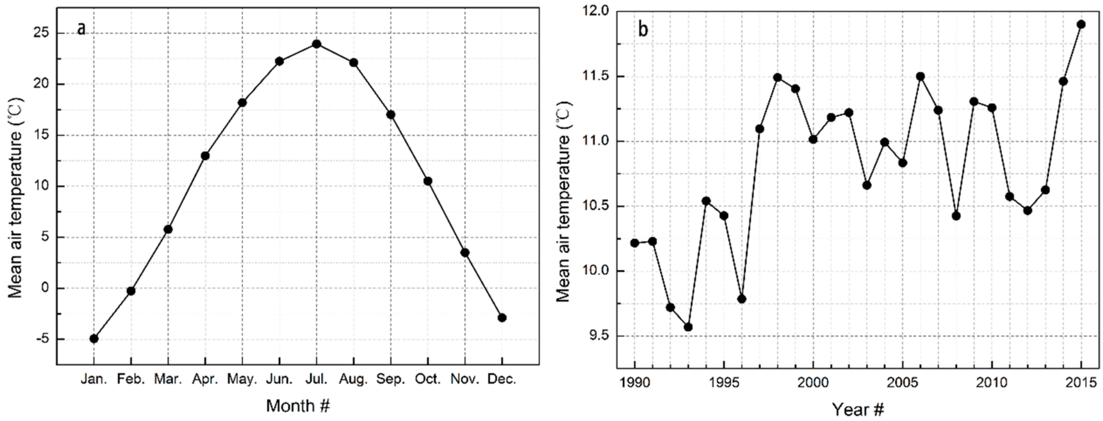

Figure S1: Fluctuations in the monthly (**a**) and yearly (**b**) mean air temperature from 1990 to 2015, where the mean value was calculated using data obtained from Yan'an city weather stations.

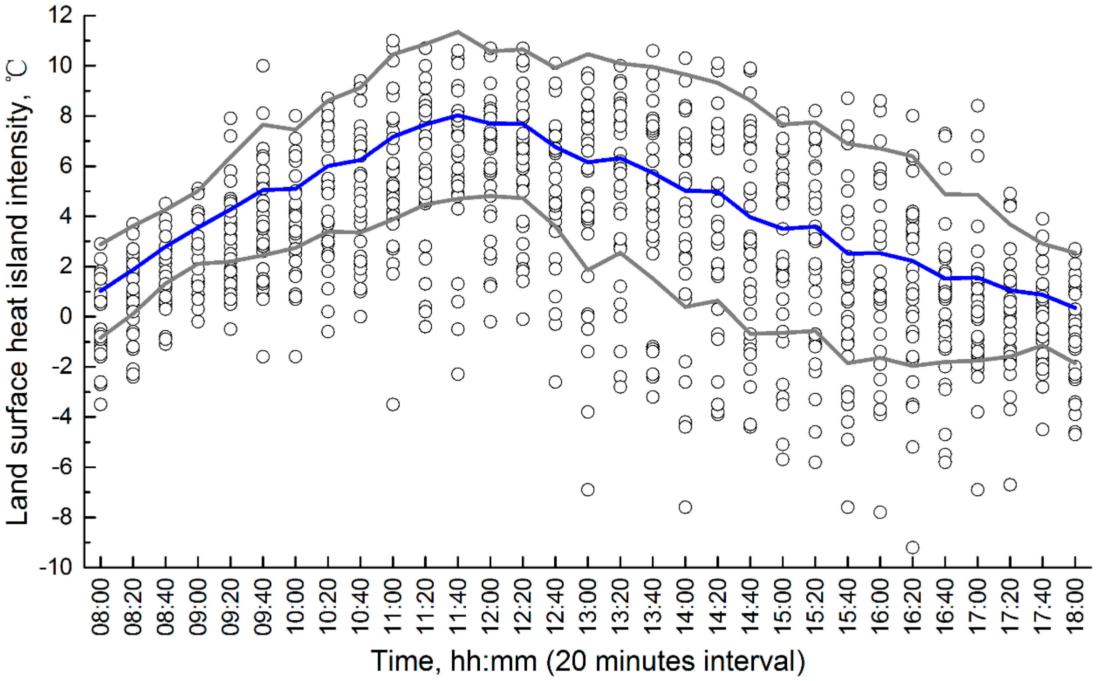

Figure S2: The 20-min variations in the land surface heat island intensity during July 2015 under sunny weather at 34 open impervious surface sites located close to various green spaces in the core urban area of Yan'an City. The mean (blue line) and standard deviation (gray line) for each 20 min period are also shown.

Table S3: The importance value of tree species in the 34 sample plots of the UGSs (%).

Order	Species	IV	Order	Species	IV	Order	Species	IV
1	PT	11.33	12	PS	3.64	22	ZJ	1.24
2	SM	10.99	13	RP	3.23	23	AM	1.17
3	SJ	9.79	14	PCa	3.21	24	AJ	1.16
4	GB	5.71	15	PU	2.85	25	PAs	0.56
5	PA	5.53	16	JF	2.61	26	PB	0.53
6	PH	5.30	17	FC	2.09	27	WS	0.39
7	JR	4.75	18	AP	2.03	28	SJv	0.34
8	PO	4.62	19	SC	1.39	29	CD	0.31
9	AV	4.42	20	SR	1.39	30	MA	0.18
10	UP	3.94	21	AG	1.24	31	PC	0.17
11	SJp	3.87						

AG: *Acer ginnala* Maxim. subsp. Ginnala; AJ: *Albizia julibrissin* Durazz; AM: *Acer mono* Maxim; AP: *Amygdalus persica* L.; AV: *Armeniaca vulgaris* Lam.; CD: *Cedrus deodara* (Roxb.) G. Don; FC: *Fraxinus chinensis* Roxb.; FS: *Forsythia suspensa* (Thunb.) Vahl f. *suspensa*; GB: *Ginkgo biloba* L.; JF: *Juniperus formosana* Hayata; JR: *Juglans regia* L.; MA: *Morus alba* L.; PA: *Picea asperata* Mast.; PB: *Pinus bungeana* Zucc. ex Endl.; PC: *Pistacia chinensis* Bunge; PCa: *Prunus cerasifera* Ehrhar f. *atropurpurea* (Jacq.) Rehd.; PH: *Populus hopeiensis* Hu et Chow in Bull.; PO: *Platycladus orientalis* (L.) Franco; PS: *Pinus sylvestris* Linn. var. *mongolica* Litv.; PT: *Pinus tabuliformis* Carr.; PU: *Pyrus ussuriensis* Maxim.; RP: *Robinia pseudoacacia* Linn.; SC: *Sabina chinensis* (L.) Ant.; SJ: *Sophora japonica* Linn.; SJp: *Sophora japonica* Linn. var. *japonica* f. *pendula* Hort.; SJv: *Sophora japonica* Linn. var. violacea Carr.; SM: *Salix matsudana* var. *matsudana* f. *pendula* Schneid.; SR: *Syringa reticulata* (Blume) Hara var. amurensis (Rupr.) Pringle; UP: *Ulmus pumila* L.; WS: *Wisteria sinensis* (Sims) Sweet.; ZJ: *Ziziphus jujuba* Mill.

The Importance value (IV) of species is defined as the average of relative density (RD), relative frequency (RF), and relative dominance (Rd) of that species and was was calculated using the following equations (S1) [80]:

$$\mathrm{IV}_i = \frac{1}{3}\left(\frac{N_{Si}}{\sum_{i=1}^{n}\frac{N_{Si}}{A_i}} + \frac{Nq_i}{n \times m} + \frac{BA_i}{\sum_{i=1}^{n} BA_i}\right) \quad \text{(S1)}$$

where N_{Si}, Number of individuals of the i species; A_i, area of all samples unites; *n*, number of the domain species (*n*=31); Nq_i, number of quadrats containing the *i* species; m, total number of quadrats (*m*=34); BA_i, Basal area of the *i* species.
